# Genome-wide annotation and expression analysis of *WRKY* and *bHLH* transcriptional factor families reveal their involvement under cadmium stress in tomato (*Solanum lycopersicum* L.)

**DOI:** 10.3389/fpls.2023.1100895

**Published:** 2023-01-25

**Authors:** Ibrahim Khan, Sajjad Asaf, Rahmatullah Jan, Saqib Bilal, Lubna, Abdul Latif Khan, Kyung-Min Kim, Ahmed Al-Harrasi

**Affiliations:** ^1^ Natural and Medical Sciences Research Center, University of Nizwa, Nizwa, Oman; ^2^ Department of Applied Biosciences, Kyungpook National University, Daegu, Republic of Korea; ^3^ Department of Engineering Technology, University of Houston, Sugar Land, TX, United States

**Keywords:** *Solanum lycopersicum*, phylogenetic analysis, heavy metal stress, expression pattern, *WRKY* and *bHLH*

## Abstract

The *WRKY* and *bHLH* transcription factors have been implicated in the regulation of gene expression during various physiological processes in plants, especially in plant stress responses. However, little information about the heavy metal-responsive *SlWRKY* and *SlbHLH* in tomato (*Solanum lycopersicum*) is available. We performed a genome-wide investigation for these two TF families in *S. lycopersicum* and determined their role in cadmium (Cd) stress tolerance. Furthermore, ortholog analysis with the *Arabidopsis* genome led to classifying *WRKY* and *bHLH* ortholog genes into nine and 11 clusters, respectively. The comparative phylogenetic analysis revealed duplication events and gene loss in *Arabidopsis* and *S. lycopersicum*, which occurred during evolution both before and after the last common ancestor of the two species. Orthologous relationships are also supported by additional evidence, such as gene structure, conserved motif compositions, and protein–protein interaction networks for the majority of genes, suggesting their similar functions. A comprehensive transcriptomics analysis revealed that both *WRKY* and *bHLH* genes were differentially expressed in response to cadmium stress as compared with control plants. A gene ontology analysis revealed that most *WRKYs* and *bHLHs* are DNA-binding essential proteins that regulate gene expression positively and negatively. Analyses of interaction networks revealed that both *WRKYs* and *bHLHs* mediate networks implicated in several stress-signaling pathways. The findings of this work may help us to comprehend the intricate transcriptional control of *WRKY* and *bHLH* genes and identify potential stress-responsive genes relevant to tomato genetic improvement. Moreover, identifying heavy metal stress-responsive *WRKY* and *bHLH* genes in *S*. *lycopersicum* will provide fundamental insights for developing new heavy metal stress-tolerant varieties of tomato crops.

## Introduction

Tomato (*Solanum lycopersicum*) is one of the most economically important cultivated crops worldwide. Its consumption increases annually due to its multiple utilization (Gerszberg et al., 2015). Despite its pivotal importance in breeding and evolutionary studies due to its fruit attractiveness (flavors, colors, shapes, and sizes), genome-wide research studies are limited. The recent release of the complete assembly of the sequenced genome of tomato allowed us to identify and characterize novel gene families genome-wide (Huang et al., 2012). In nature, plants, including tomatoes, constantly face various biotic and abiotic stresses. Heavy metal excess is one of the plants’ most destructive abiotic stresses (Li et al., 2022). Over the last few decades, the rapid increase in human population and anthropogenic activities such as urbanization, industrialization, and modern agricultural practices has resulted in an increase in the quantity of heavy metal toxicity in the surroundings, which causes chronic toxicity to living things (Khalil et al., 2021). Because of pesticides, fertilizers, municipal waste, smelting industries, and metalliferous mining, large areas of land have become contaminated with heavy metals (Singh et al., 2016). Although numerous heavy metals exist naturally in variable amounts in the earth’s crust, the issue arises when they are released into the environment in significant amounts as a result of natural or anthropogenic activities (Duruibe et al., 2007). Based on their density (>5 g/cm^3^), the 53 d-block elements have been classified as heavy metals (Dutta et al., 2018). Plant cells require only 19 elements, which are further categorized into two groups based on the essentiality for completing a plant’s life cycle, i.e., macro- and micro-elements (Subbarao et al., 2003). Macro-elements include C, H, O, N, Mg, S, P, Cd, and K, while Ni, Zn, Cu, Cl, Fe, Mn, Mo, B, Co, and Br are micro-elements. Different physiological and biochemical processes in plants are significantly influenced by these macro and micro heavy metals, such as the biosynthesis and metabolism of nucleic acids, proteins, and chlorophyll; sugar metabolism; and nitrogen fixation (Rout and Sahoo, 2015). For instance, zinc (Zn) is considered as a multipurpose micro-element due to its capacity to bind to >300 enzymes and 200 transcription factors (TFs) as a co-factor to activate these enzymes and TFs to maintain auxin metabolism, cell membrane integrity, and reproduction (Gondal et al., 2021), and a sufficient supply of nickel (Ni) is essential for several physiological processes including seed germination, optimal vegetative growth and reproductive development, and improved crop yield and quality (Khalil et al., 2021). However, high concentrations and bioavailable forms of heavy metals may adversely affect plant health, eventually resulting in cell death. Some heavy metals such as mercury (Hg), aluminum (Al), cadmium (Cd), lead (Pb), and chromium (Cr) are very toxic at very low concentrations and produce severe toxicity symptoms in plants including biomass accumulation, restriction of growth and photosynthesis, chlorosis, disturbed water balance, and nutrient absorption, all of which eventually lead to plant death (Kalaivanan and Ganeshamurthy, 2016). Heavy metal stress is estimated to be one of the major causes of global crop yield reduction. This situation has deteriorated due to a disruption in the balance between crop productivity and population increase (Shahid et al., 2015). It is therefore crucial to comprehend how plants respond to these stresses to develop novel quantitative and qualitative strategies for enhancing crops. Heavy metal stress signals trigger various physiological and biochemical pathways involving many genes to develop strategies enabling them to cope with the adverse effects of heavy metal toxicity (Singh et al., 2016). In plants, many TFs are involved in regulating the expression of various stress-responsive genes cooperatively or separately, and the genes responsible for TFs can do wonders in modifying the crops such that they can adapt in heavy metal-rich soils (Khan et al., 2021a; Manzoor et al., 2022). Regulatory genes encode biochemical substances such as sugars, alcohols, and amines, which serve an important function in plants against heavy metal stress. Several studies have shown that a single TF can influence the expression of numerous genes by selectively binding to the cis-acting element in the promoter region of its target genes and through a protein–protein or domain–domain interaction that facilitates TF oligomerization with other regulatory proteins (Shiu et al., 2005; Nakashima et al., 2009). From more than 80 different TF families, only a few, such as *WRKY*, *bHLH*, *GRAS*, *MYB*, *AP2*/*ERF*, *Dof*, *bZIP*, and *DREB*, with crucial roles in heavy metal stress responses have been widely studied, and much remains to be discovered about the essential regulatory roles of diverse plant TFs (Khan et al., 2021b). Only a few tomato metal-tolerance proteins (MTPs) have been studied and functionally characterized in tomato (El-Sappah et al., 2021). Therefore, it is necessary to identify genes that are potentially involved in heavy metal stress tolerance. In this regard, the current study has emphasized identifying and characterizing two large and very important families of TFs, i.e., *WRKY* and *bHLH* gene families, in tomato plants. The detailed composition and mode of action of both *WRKY* and *bHLH* TFs are well-explored (Heim et al., 2003; Chen et al., 2019; Hao et al., 2021; Wani et al., 2021; Guo et al., 2022). Here, we will focus on the comparative genomic studies and functional roles of *WRKY* and *bHLH* TFs in tomato, particularly in heavy metal defense.

The *WRKY* TFs are also called jack-of-all-trades because they regulate many developmental and physiological processes, such as seed dormancy and germination, seed development, root formation, plant growth, senescence, trichome morphogenesis, and response to various biotic and abiotic stress factors (Ülker and Somssich, 2004; Rushton et al., 2010; Mao et al., 2011; Huang et al., 2012; Imran et al., 2019; Guo et al., 2022). To date, 81 *WRKY* (Zhao et al., 2021) and 161 *bHLH* (Riaño-Pachón et al., 2007) TFs have been identified from the entire tomato genome through genome-wide analysis. However, very little is known about the specific physiological roles of most *WRKY* and *bHLH* genes in response to heavy metal stress in tomato plants. Here, we attempted to predict the role of these uncharacterized TFs in response to heavy metal stress by comparative genomic analysis with the most extensively studied plant species, *Arabidopsis thaliana*.

The current study will offer evolutionary and functional roles of *WRKY* and *bHLH* genes in tomato, which can open new windows for future studies to generate stress-resistant crop cultivars. Several *in-silico* analyses, such as subcellular locations, phylogenetic relationships, gene structure, conserved domains, gene ontology, protein–protein interactions, co-expression patterns, and expression pattern analysis and functional annotations, were utilized to get insights into the physiological roles of *WRKY* and *bHLH* TFs in tomato. To determine the specific regulatory role of *WRKY* and *bHLH* genes in various physiological processes, their orthologous and paralogous pairs have also been identified, and the authenticated RNA-seq data with reverse transcription-quantitative PCR (qRT-PCR) were used to evaluate their expression and to elucidate their functional roles.

## Materials and methods

### Identification of *bHLH and WRKY* proteins

The nucleotide and amino acid sequences of the AtWRKY and AtbHLH proteins of *Arabidopsis* were downloaded from The Arabidopsis Information Resource (TAIR, https://www.arabidopsis.org/). Nucleotide sequences of the *SlWRKY* and *SlbHLH* proteins of *S*. *lycopersicum* were retrieved from Phytozome (https://phytozome-next.jgi.doe.gov/) and Sol Genomics Network (SGN, https://solgenomics.net/), while their corresponding protein sequences were downloaded from the Plant Transcription Factors Database (PlantTFDB, http://planttfdb.gao-lab.org/). The subcellular localizations of *SlWRKY* and *SlbHLH* proteins were predicted using CELLO (http://cello.life.nctu.edu.tw/) (**Table S1**). The protein sequences of putative *SlWRKY* and *SlbHLH* members were subjected and analyzed with Expasy ProtParam (https://web.expasy.org/protparam) and SGN (https://solgenomics.net/) to determine their theoretical isoelectric point (p*I*), molecular weight (Mw), and number of amino acids (AAs).

### Phylogenetic analysis of *WRKY* and *bHLH* genes in tomato and among tomato and *Arabidopsis*

Neighbor-joining (NJ) phylogenetic trees of the 72 *Arabidopsis* with 81 *S. lycopersicum WRKY* and 153 *Arabidopsis* with 161 *S. lycopersicum bHLH* proteins were constructed using the software MEGA 11 with 1,000 replicates for bootstrap (BS) analysis for statistical reliability to compare the evolutionary relationships of *WRKY* and *bHLH* TFs across the species. We further performed NJ phylogenic tree analysis among *WRKY* and *bHLH* protein sequences in *S. lycopersicum* to test the reliability of the results.

### Gene structure and motif composition analysis

The CDS and genomic sequences of the *WRKY* and *bHLH* of *Arabidopsis* and *S. lycopersicum* were retrieved from TAIR (https://www.arabidopsis.org) and Phytozome (https://phytozome-next.jgi.doe.gov/), respectively. The Gene Structure Display Server (GSDS, http://gsds.gao-lab.org/) web tool was used to analyze the structure of the members of the *WRKY* and *bHLH* gene families and to detect their exon–intron organization, by aligning the CDS sequences with genomic sequences. To identify the conserved motifs in these protein sequences, the Multiple EM for Motif Elicitation (MEME, https://meme-suite.org/meme/tools/meme) online server was used with the following parameters: number of repetitions, any; maximum number of motifs, 15; and optimum motif width set to ≥6 and ≤200 amino acid residues.

### Chromosomal location and gene duplication analysis

In order to determine the physical locations of the *SlWRKY* and *SlbHLH* genes in the *S. lycopersicum* genome, the starting and ending positions of all the identified genes on each chromosome were obtained from the Solanaceae family database (Sol Genomics Network). The MapInspect software (http://mapinspect.software.informer.com/) was used to map the physical locations of the *SlWRKY* and *SlbHLH* genes on their respective chromosomes. The duplicate chromosomal blocks were retrieved using the Plant Genome Duplication Database (PGDD, accessible at http://chibba.agtec.uga.edu/duplication/), and the *WRKY* and *bHLH* genes within the duplication block were identified. This allowed us to find duplicate *S. lycopersicum WRKY* and *bHLH* genes (Lee et al., 2012). Genes separated by five or fewer gene loci in a range of 100 kb distance were considered to be tandem duplicates, and those which were co-paralogs and located within duplicated chromosomal blocks in multiple locations as a result of duplication and chromosome rearrangement and shared >90% sequence identity were considered as segmental duplicates (Wei et al., 2007). The PGDD, a public web service database, was used to identify and characterize the genes in terms of intra- or interplant genomic syntenic relationships.

### Growth conditions, Cd stress treatment, and RNA extraction

In the current study, tomato seeds (*S. lycopersicum* cv. Yegwang) were used. First, the seeds were surface-sterilized with 10% hypochlorous acid and 70% ethanol and washed with autoclaved distilled water to remove the impurities. The soaked seeds were germinated on hygiene filter paper in an incubator at 30°C in dark conditions. After successful sprouting, the seeds were planted in plastic pots in a greenhouse. The greenhouse temperature was kept constant at 28°C ± 2°C, 55% ± 5% relative humidity, and 16-h light/8-h dark photoperiod. The experiment was conducted utilizing three groups of plants: a) distilled water-treated plants (control plants), b) 1-mM Cd-treated plants, and c) 2-mM Cd-treated plants. After every 3 days, the plants were treated with their respective treatments under the same growth conditions. Four replicates were prepared per treatment. Leaf samples were collected randomly and immediately placed in liquid nitrogen and stored at –80°C in a fridge until analysis.

### RNA extraction and sequencing

RNA was isolated using the RNeasy Plant Mini Kit (QIAGEN, Hilden, Germany). The quantity of total RNA was adjusted to 10 μg, and the NanoDrop ND-1000 (Thermo Scientific, Waltham, MA, USA) was used to evaluate the quality. Sequencing and analysis of libraries from three different biological replicates of each treatment were performed. The Illumina HiSeq 2000 platform was utilized to perform library construction according to the described approach (Liao et al., 2015), resulting in single-end reads with 51 bp. To identify variations in gene expression regulation between the cadmium-treated and non-treated plants, a computational pipeline of optimized tools was employed. For trimming and quality check, Trim Galore (Krueger, 2012) and FastQC (Andrews, 2010) were used, respectively. To align the reads to the reference genome, HISAT2 (Sirén et al., 2014) was used before the read count quantification by using Feature Count (subread_v2.0.2). The R software using the DESeq2 (Love et al., 2014) package was used for differential gene expression analysis.

### Prediction of the protein–protein interaction network

The STRING protein interaction database version 11.5 (https://string-db.org) was used to identify the interacting protein networks and functional annotations.

### Gene ontology-based functional annotation analysis

The online software agriGO analysis toolbox (http://bioinfo.cau.edu.cn/agriGO/) was used to enrich gene ontology (GO) categories (Du et al., 2010) using the TopGO “elim” algorithm (Alexa et al., 2006) covering the following characteristics: biological processes, molecular processes, and cellular processes.

### Reverse transcription-quantitative PCR

For qRT-PCR analysis, approximately 10 highly expressed genes (5 *WRKY* and 5 *bHLH*) from RNA-seq data were selected to authenticate the RNA-seq results. The Primer3 (https://bioinfo.ut.ee/primer3-0.4.0/) program was used to design primers for each selected gene as listed in **Table S3**. The standard cDNA was produced using PCR Biosystems’ qPCRBIO cDNA Synthesis Kits after total RNA was diluted to a final concentration of 100 ng/μl, and transcript quantification was done as reported previously (Asaf et al., 2022). Actin (a housekeeping gene) was used as an internal control to normalize gene expression, and the comparative ΔΔCt method of qRT-PCR was utilized to calculate the expression level of the genes in control plants in comparison with Cd-treated ones.

## Results

### Identification of the *WRKY* and *bHLH* families in *Solanum lycopersicum* and *Arabidopsis* genomes

In order to systematically identify and analyze *WRKY* and *bHLH* genes in the tomato genome, their sequences were downloaded as described above. Finally, a total of 81 and 161 non-redundant putative *WRKY* and *bHLH* genes were confirmed by their specific domain using SMART (http://smart.embl-heidelberg.de) (**Table S1**). Subsequently, the downloaded gene sequences were compared with their expressed sequence tag (EST) sequences in PlantTFDB (http://planttfdb.gao-lab.org). *Arabidopsis WRKY* and *bHLH* genes were obtained from the TAIR (https://www.arabidopsis.org) and PlantTFDB (http://planttfdb.gao-lab.org). A total of 72 *WRKY* and 161 *bHLH* annotated TFs were extracted from these sources (**Table S1**).

### Phylogenetic analysis and recognition of *WRKY* and *bHLH* families in *Solanum lycopersicum*

**Figure 1:**
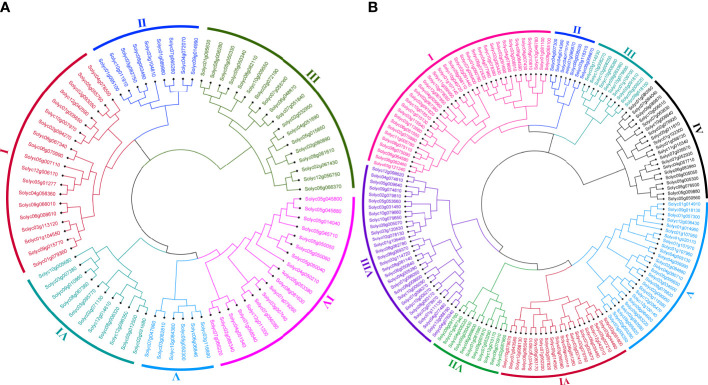
**(A)** The phylogenetic tree of 81 *Solanum lycopersicum WRKY* TFs constructed by the neighbor-joining (NJ) method using the MEGA 11 software with 1,000 bootstrap replicates. The six major phylogenetic groups are marked as I to IV, respectively. **(B)** The phylogenetic tree of 161 *S. lycopersicum bHLH* TFs constructed by the NJ method using the MEGA 11 software with 1,000 bootstrap replicates. The eight major phylogenetic groups are marked as I to VIII, respectively.

**Figure 2:**
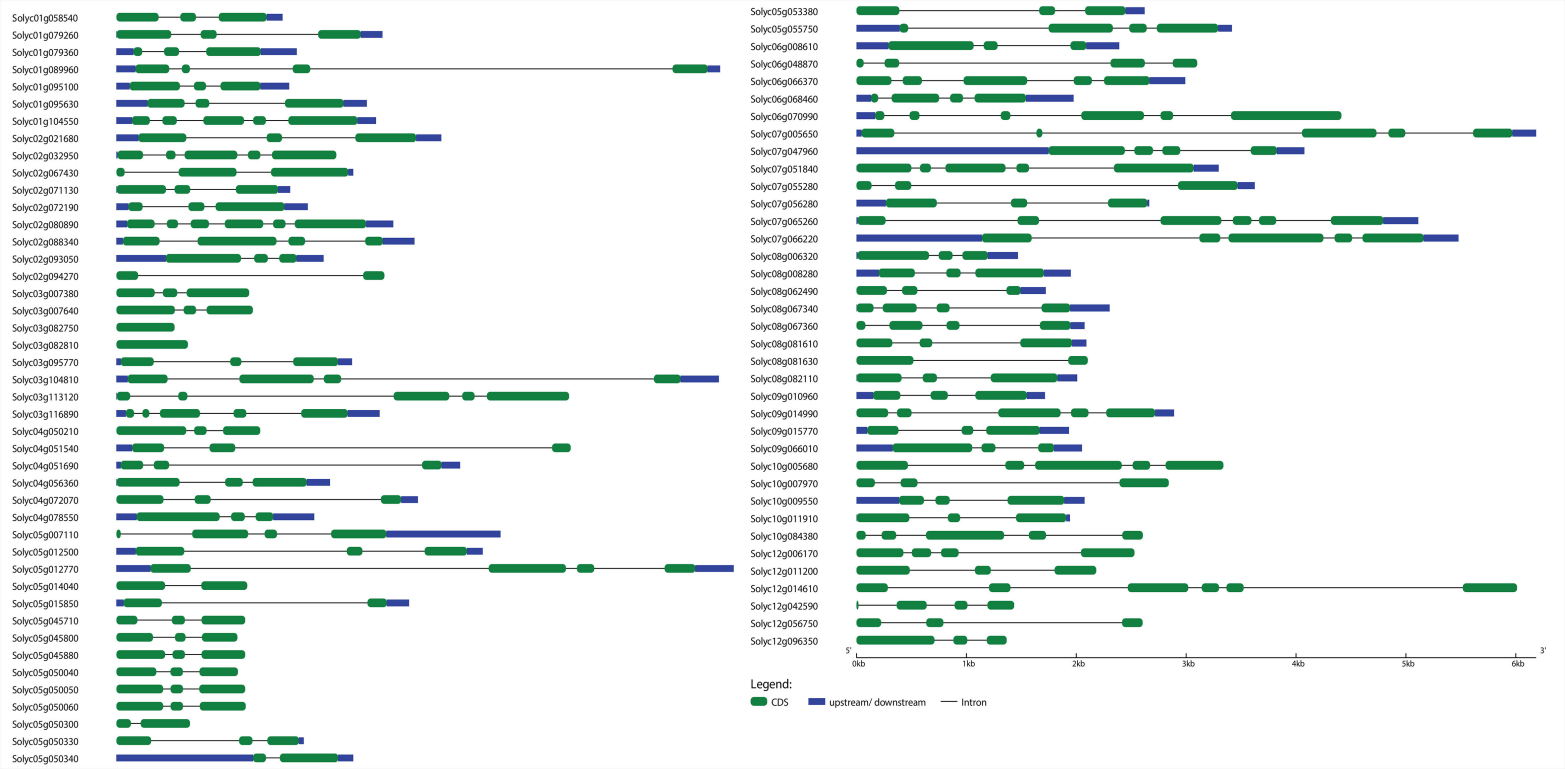
Exon–intron structure analysis of *Solanum lycopersicum WRKY* genes was performed by the GSDS database. The blue boxes indicate upstream/downstream, the green boxes indicate exons, and the black lines indicate introns.

**Figure 3:**
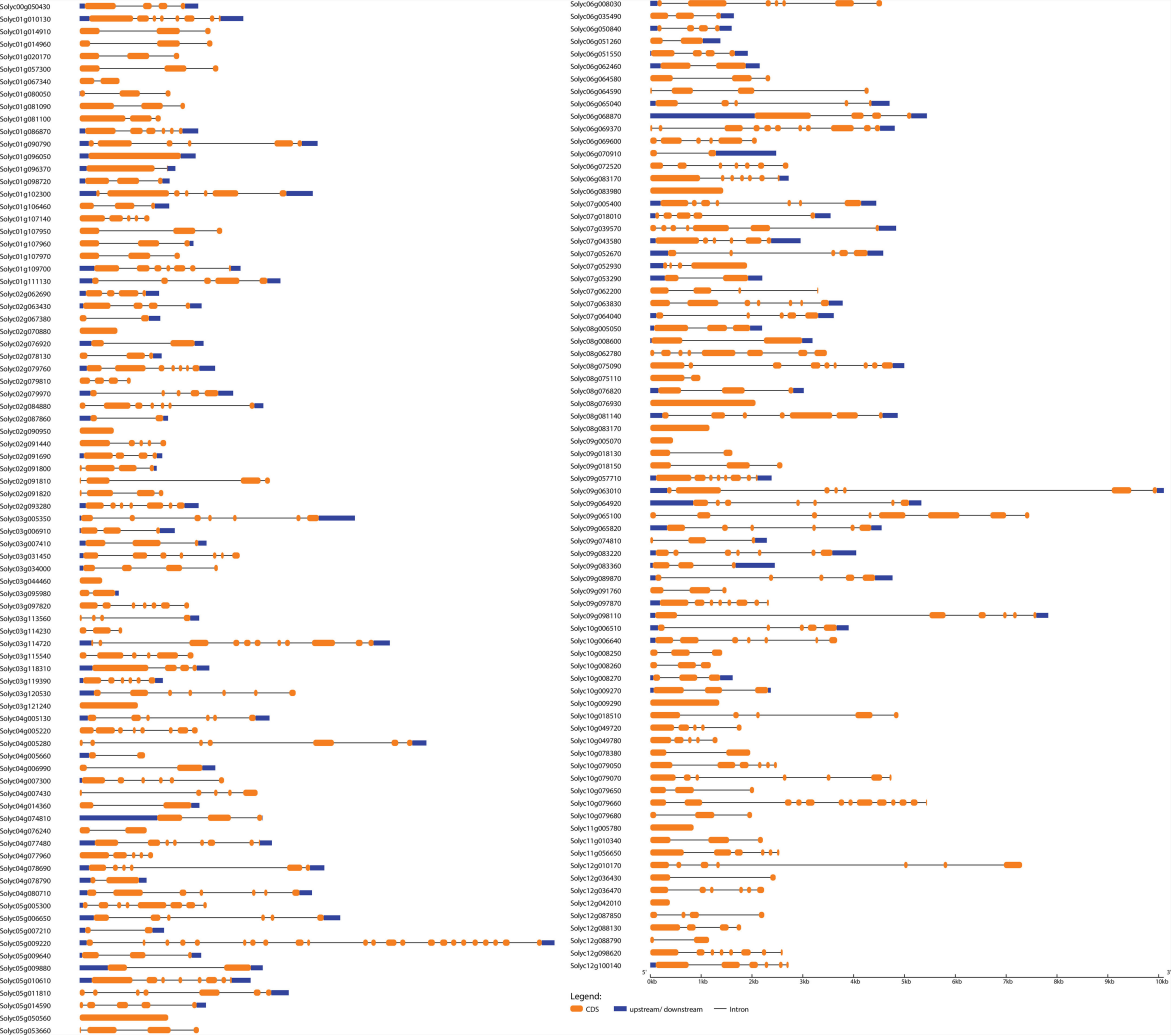
Exon–intron structure analysis of *S. lycopersicum bHLH* genes was performed by the GSDS database. The yellow boxes indicate upstream/downstream, the blue boxes indicate exons, and the black lines indicate introns.

In order to determine the evolutionary and phylogenetic relationship among the *S. lycopersicum WRKY* as well as among *bHLH* proteins, unrooted neighbor-joining phylogenetic trees were built by a multiple sequence alignment of their amino acid sequences using the ClustalW program. As shown in **Figure 1A**, *WRKY* TFs of *S. lycopersicum* were divided into six main groups (group I to group VI). Notably, the maximum number, i.e., 19 (23.4%) of the *WRKY* members, was found in group I, followed by groups III and IV with 18 (22.2%) members. The least number, i.e., 6 (7.4%) of the *WRKY* members, was found in group V. Similarly, *bHLH* TFs were divided into eight main groups (group I to group VIII) based on the phylogenetic tree. The maximum number, i.e., 32 (19.9%) of the *bHLH* members, was found in group I, followed by group VIII with 30 (18.6%) and group V with 26 (16%) members. The least number, i.e., 7 (4%) of the *bHLH* members, was found in group II (**Figure 1B**). Furthermore, most of the members from the same phylogenetic group have the same number of exons and conserved exon–intron structure (**Figures 2**, **3**). For instance, in groups III and IV, 18 (75%) and 14 (78%) of the *SlWRKY* members have three exons, respectively. On the other hand, in the phylogenetic tree of *SlbHLH* TFs, group VIII contained the majority of the genes with 6–13 exons. Our results also showed that most of the evolutionarily related members have similar motif compositions and the same subcellular locations (**Figures S1**, **S2** and **Table S1**). The similarity in these features may be related to their specific physiological functions in tomato cells. The high bootstrap values (95%–100%) indicate a strong phylogenetic relationship between some pairs of the *SlWRKY* TFs. For instance, Solyc05g050050–Solyc05g050060, Solyc01g058540–Solyc04g051540, and Solyc05g050330–Solyc05g050340 pairs of *SlWRKY* genes showed a paralogous relationship, supported by 99% of bootstrap values (**Figure S3**), while some pairs of *SlbHLH* TFs, i.e., Solyc01g014910–Solyc09g018130, Solyc01g020170–Solyc01g107970, Solyc03g119390–Solyc12g036470, Solyc09g089870–Solyc10g006510, and Solyc10g006510–Solyc08g075090, had 100% bootstrap values as shown in **Figure S4**.

### Phylogenetic analysis of *WRKY* and *bHLH* genes in *Arabidopsis* and *Solanum lycopersicum*

To identify the evolutionary history and phylogenetic relationship between the *SlWRKY* (81 members) and *AtWRKY* (72 members) and between the *SlbHLH* (161 members) and *AtbHLH* (153 members), unrooted NJ phylogenetic trees were derived from the alignment of the amino acid sequences of these TFs. The tree inferred from the *WRKY* TFs of *Arabidopsis* and *S. lycopersicum* organized *WRKY* into nine main groups (group I to group IX) (**Figure 3A**). Among the nine main groups, group IX is the largest one containing 29 members, followed by group I with 24 members and group IV and group VIII with 20 members, while group VI was the smallest which contained only nine members. For *bHLH* TFs, 11 groups (group I to group XI) were defined based on their constructed phylogenetic tree. Group V is the largest one with 50 *bHLH* members, followed by group I with 45 members and group III with 41 members, while group II has the least number (only 10) of members (**Figure 3B**).

### Insights from the paralogy and orthology relationship

Genes that descended from a common ancestral gene are likely to have the same functions. Therefore, homological analysis is widely used to predict the function of an uncharacterized gene (Dai et al., 2020). Paralogs are homologous genes evolved by duplication of an ancestral gene which can keep the same or diverge to a different functional role (Gabaldón and Koonin, 2013), and orthologous relationships between genes from different species can reflect the same evolutionary and functional niche in different species (Fang et al., 2010). The tree topology and the organization of most of the groups and subgroups resembled those from the *S. lycopersicum* and *Arabidopsis* individual trees. The trees presented in **Figures 1**, **3** identified putative paralogs and orthologs. A total of 27 and 50 orthologous pairs of *WRKY* and *bHLH* genes, respectively, were identified across the species. Most *S. lycopersicum* and *Arabidopsis* paralogous genes observed in **Figures 3A, B** were already displayed as paralogs in the respective trees (**Figures 1A, B**), for instance, Solyc01g058540–Solyc04g051540, Solyc05g050330–Solyc05g050340, and Solyc05g050050–Solyc05g050060 pairs of the *SlWRKY* TFs and Solyc09g089870–Solyc10g006510, Solyc08g075090–Solyc08g075110, and Solyc01g020170–Solyc01g107970 pairs of the *SlbHLH* TFs. The exception of a few TFs for not displaying in the same paralogous pairing in both individual and combined trees may be due to the existence of an apparent ortholog from a different species, providing better support for the new location, e.g., Solyc12g014610–AT1G13960 and Solyc02g021680–AT2G40740 pairs of group VI of the combined phylogenetic tree of *SlWRKY* and *AtWRKY* TFs. It is worth noting that most of the described phylogenetic groups and subgroups were supported by additional evidence, such as gene structure, conserved motifs, gene ontology, protein–protein interaction, and similarity in physiological functions of most of the characterized genes.

### Phylogenetic-based gene function prediction

Analysis of the orthologous and paralogous relationships is widely used to predict the function of an uncharacterized gene because the genes that descended from a common ancestral gene are likely to have the same functions (Dai et al., 2020). When comparing multigene families of different species, it is frequent and interesting to find multiple genes in one species that are orthologous to a single gene in the other, indicating recent duplications specific to the former. The orthologous genes with similar sequences and the same expression patterns in various organisms suggest a possible case of functional redundancy. There are no detailed data on the specific physiological roles of most *S. lycopersicum* genes. Since both *WRKY* and *bHLH* TFs have important roles in several important major developmental and physiological processes and responses to different environmental stimuli, it is critical to conduct extensive research on the *WRKY* and *bHLH* gene families in *S. lycopersicum*. The comparative genome-wide study of *S. lycopersicum WRKY* and *bHLH* with *WRKY* and *bHLH* TFs of *Arabidopsis*, which is the most extensively studied plant, will allow us to predict their physiological functions. For instance, AT1G01260 is involved in negatively regulating jasmonic acid during leaf senescence and making an orthologous pair by a strong BS score with the Solyc01g096050 gene of the *bHLH* family. Similarly, AT1G26945 is involved in ABA and salt stress responses resulting from an orthologous pair by a strong BS score with Solyc06g070910 of the *bHLH* family (Hao et al., 2021). So, based on these orthologous relationships, we can predict the function of Solyc01g096050 and Solyc06g070910 genes in *S. lycopersicum*.

### Insights into the gene structures and conserved motifs of *WRKY* and *bHLH* genes

**Figure 4:**
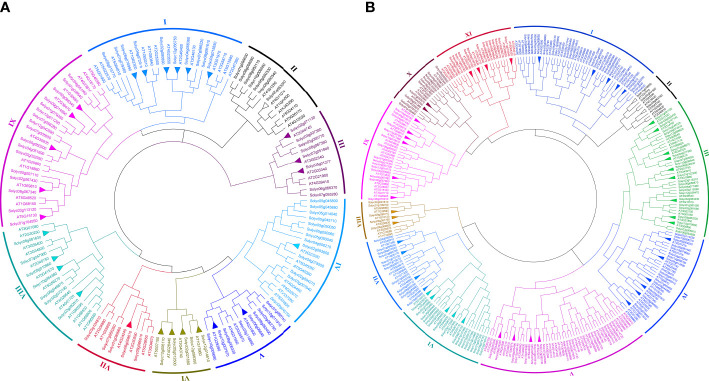
**(A)** Joined phylogenetic tree constructed from an alignment of 81 *Solanum lycopersicum* (*SlWRKYs*) and 72 *Arabidopsis thaliana* (*AtWRKYs*) protein sequences by the NJ method with bootstrapping (1,000 replicates) using the MEGA 11 software. The resulting nine groups are shown in different colors. **(B)** Joined phylogenetic tree constructed from an alignment of 161 *S. lycopersicum* (*SlbHLHs*) and 153 A*. thaliana* (*AtbHLHs*) protein sequences by the NJ method with bootstrapping (1,000 replicates) using the MEGA 11 software. The resulting 11 groups are shown in different colors.

To further understand the evolutionary patterns and gene duplication events, structural and compositional analyses of genes can be used as supporting evidence (Khan et al., 2021a). The *WRKY* and *bHLH* genes’ exon–intron patterns and conserved motifs were studied to provide insight into the evolution of these two gene families in the *S. lycopersicum* genome. The results showed that the number of exons and introns in *WRKY* genes ranged from 6 to 1 and 0 to 5, respectively, whereas the number of exons and introns in *bHLH* genes ranged from 23 to 1 and 0 to 22, respectively (**Figures 2**, **4**). The majority of the genes (45 *WRKY* and 35 *bHLH*) from both families have three exons and two introns. Genes belonging to the same group of phylogenetic trees almost had parallel structures apart from a few genes. Among the *SlWRKYs* and *SlbHLHs*, Solyc07g005650 and Solyc09g063010 possess the longest structures, respectively. Among all *SlbHLHs*, a few genes have a complex structure, such as Solyc05g009220, Solyc10g079660, and Solyc03g114720. The exon–intron arrangement of the *SlbHLH* genes showed that there are exons lost or gained during the evolution process of the *S. lycopersicum* genome.

The MEME software was used to recognize conserved motifs to better understand the divergence and possible function of the *WRKY* and *bHLH* gene families (**Figures S1**, **S2**). Like gene structure, the motif distributions were similar within the same phylogenetic group. For instance, most of the members of group IV of the individual phylogenetic tree of *SlWRKY* have eight conserved motifs, and most of the members of group V of the *SlbHLH* individual tree have seven conserved motifs. The aqua blue-colored motifs were uniformly found in almost all of the *WRKY* and *bHLH* TFs, so these motifs, i.e., DPSIVITTYEGEHNH and KTDKASMLDEAINYIKELQKQ, significantly represented the conserved *WRKY* and *bHLH* domains, respectively. We did not identify a potentially conserved motif (aqua blue) in only two members (Solyc10g008250 and Solyc10g008270) of the *bHLH* family possibly due to lack of homology, sequence repetition, rearrangements, or disruption of alignment (Gribskov, 2019). These results of the gene structures and conserved motifs suggested that TFs clustered in the same group might be meaningful for gene evolution and their physiological roles.

### Chromosomal location and gene duplication analysis

**Figure 5:**
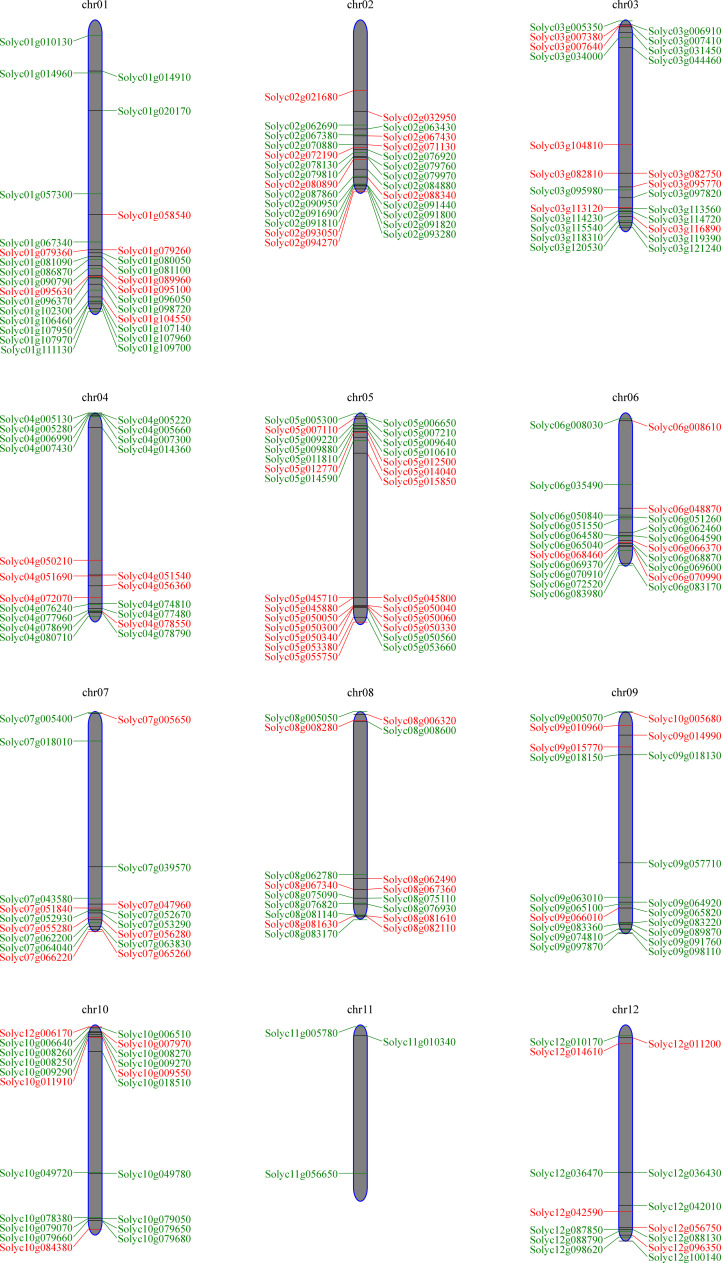
Chromosomal localizations of *SlWRKY* and *SlbHLH* genes on the 12 chromosomes of *Solanum lycopersicum*.

We map the chromosomal locations of the *SlWRKY* and *SlbHLH* genes and illustrate that majority of the *SlWRKYs* and *SlbHLHs* are clustered at the chromosomal ends. The 81 *SlWRKY* genes are distributed in all *S. lycopersicum* chromosomes except chromosome 11. As represented in **Figure 5**, most of the *WRKY* genes (19.7%) were located on chromosome 5, followed by chromosome 2 with 11.1% and chromosomes 3 and 8 with 9.8% of *WRKY* genes. In *S. lycopersicum*, out of 161, 160 *bHLH* genes were mapped from chromosomes 1 to 12, while the precise location of only one *SlbHLH* gene could not be determined. Chromosome 1 contains the highest number (13.7%) of *bHLH* genes, followed by chromosome 2 with 11.2% and chromosomes 3, 6, and 10 with 10% of *bHLH* genes, while chromosome 11 contains only 1.8% of the genes.

**Figure 6:**
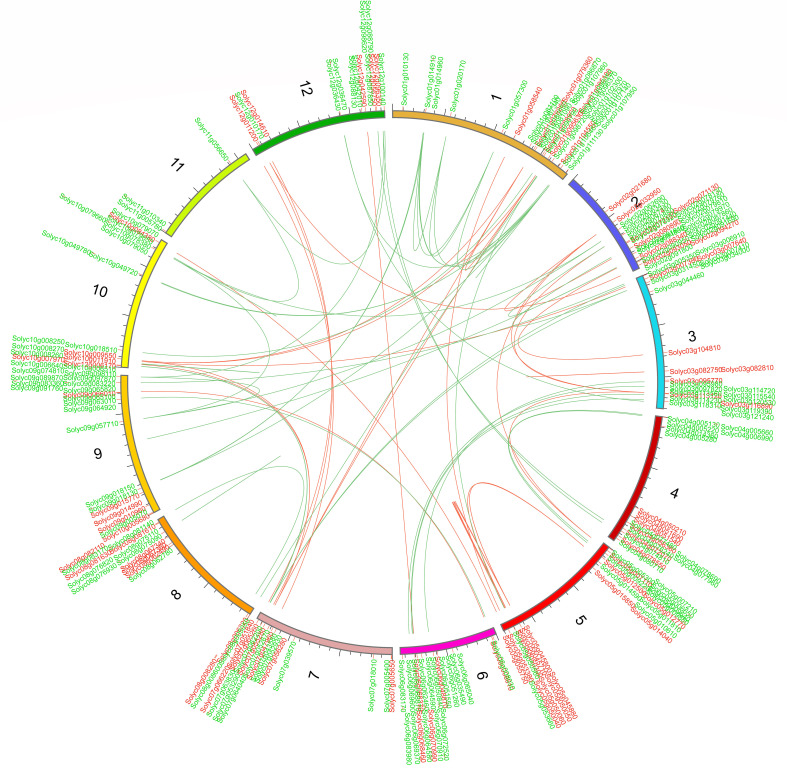
Chromosomal positions and interchromosomal groups of duplicated *SlWRKY* and *SlbHLH* gene pairs were mapped on the 12 *Solanum lycopersicum* chromosomes (Chr1–Chr12). The red and green lines represent the segmental or tandem duplication network zones among *WRKY* and *bHLH* genes, respectively.

We further determined the tandem duplications of both *WRKY* and *bHLH* genes along the 12 *S. lycopersicum* chromosomes. Tandemly duplicated genes were characterized as an array of two or more homologous genes separated by 100 kb (Khan et al., 2021a). The gene duplication study showed that more than 50% of *SlWRKY* and *SlbHLH* genes were tandemly duplicated. As shown in **Figure 6**, approximately 25 *SlWRKY* genes were tandemly duplicated unevenly on distinct chromosomes. Chromosome 2 had the most with eight *SlWRKY* gene pairs, followed by chromosome 5 with six *SlWRKY* gene pairs. Only one *SlWRKY* gene pair was discovered on chromosome 9, and chromosomes 8 and 11 had no *WRKY* gene pairs, while the Solyc05g045800 (*SlWRKY67*) and Solyc05g045710 (*SlWRKY65*) genes mapped on chromosome 5 had two pairs. The findings showed that segmental duplications assisted in the expression of *SlWRKY* genes. Similarly, approximately 28 *SlbHLH* gene pairs were discovered on distinct chromosomes in the *S*. *lycopersicum* genome. The maximum number of tandemly duplicated SlbHLH genes on chromosome 1 was 13, showing that a large number of *SlWRKY* genes on chromosome 1 were partly attributable to tandem gene duplication events.

### Protein interaction networks and functional annotations

**Figure 7:**
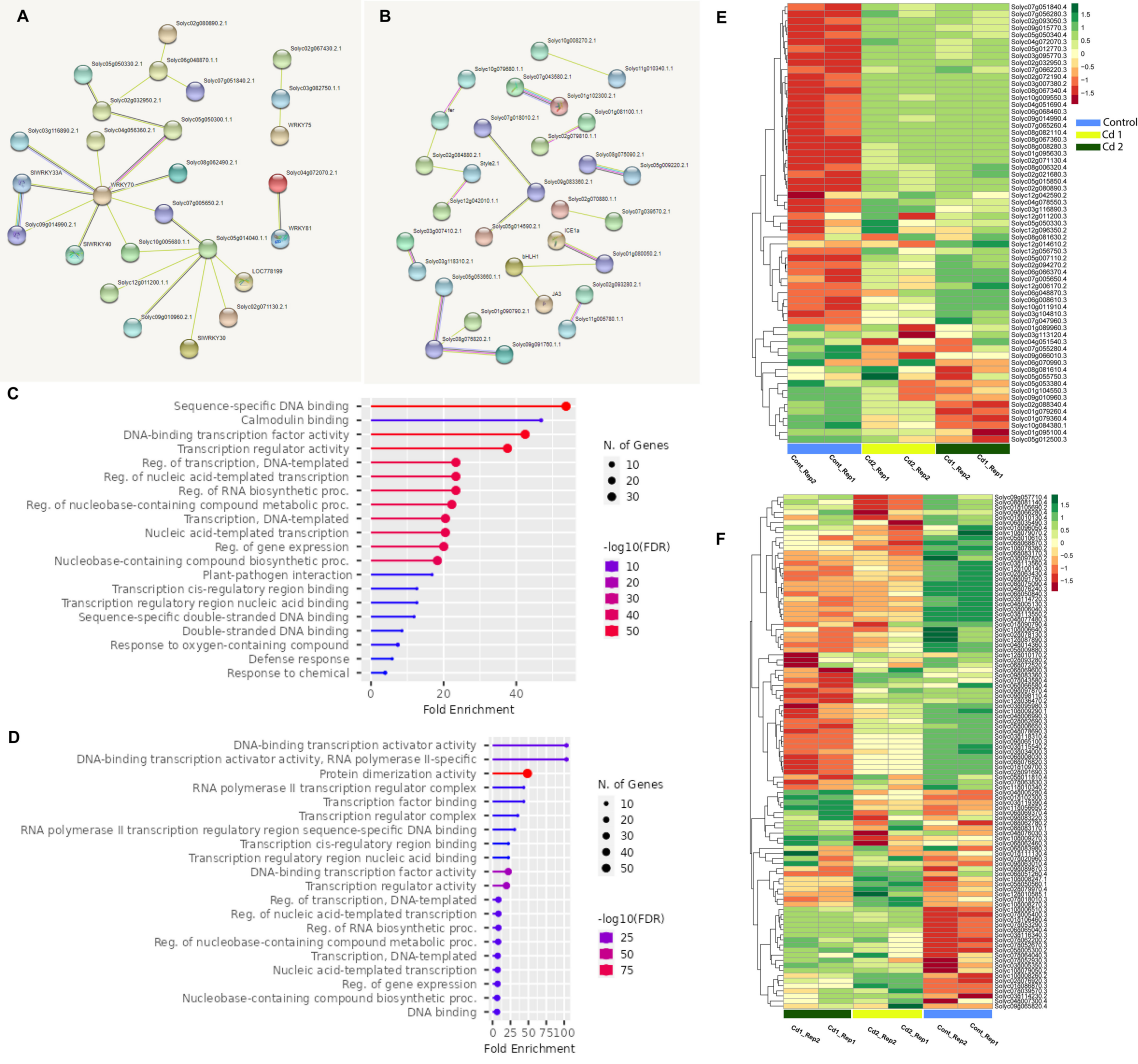
**(A)** Protein–protein association network of the *SlWRKY* genes based on their available information. The online tool STRING was used to predict the entire network. Different line colors represent the type of evidence for the associations, which are shown in the legend. **(B)** Protein–protein association network of the *SlbHLH* genes based on their available information. The online tool STRING was used to predict the entire network. Different line colors represent the type of evidence for the associations, which are shown in the legend. **(C)** Gene ontology (GO) enrichment of the *SlWRKY* genes. The size of the circles represents the number of genes in each category. **(D)** GO enrichment of the *SbHLH* genes. The size of the circles represents the number of genes in each category. Heatmap showing the expression patterns of transcriptome-wide differentially expressed **(E)** *SlWRKY* and **(F)** *SlbHLH* genes. The expression levels are quantified as log_2_ FPKM. Red and green colors indicate up- and downregulated, respectively. Two biological replicates were used for the control, 1 mM of Cd stress, and 2 mM of Cd stress.

Some *WRKY*, as well as bHLH proteins of *S. lycopersicum*, regulate the transcription process of the target genes when they form homodimer or heterodimer complexes to determine the DNA binding sites, and as a result, protein–protein interactions are critical in gene expression. The protein sequences in FASTA format were submitted to the STRING server. The protein–protein interaction network of differentially expressed proteins was constructed with the default setting. The retrieve included networks of the TFs (*WRKY* and *bHLH*), which highlight several hub proteins (**Table S2**). *WRKY* proteins that have co-expression supported by a high confidence score (0.700) include *WRKY70* (Solyc03g095770), which form a network with *SlWRKY33A* (Solyc06g066370), Solyc09g014990, Solyc03g116890, Solyc05g050300, and *SlWRKY40* (Solyc06g068460), while Solyc05g014040 has co-expression with Solyc07g005650 and Solyc09g010960 (**Figure 7A**). In the *bHLH* family, the TFs with a higher co-expression score include Solyc07g043580–Solyc01g102300, Solyc08g075090–Solyc05g009220, and ICE1a (Solyc06g068870)–Solyc08g005050, while Solyc08g076820 has co-expression with Solyc05g053660 and Solyc09g091760 (**Figure 7B**).

### Gene ontology analysis of *SlWRKY* and *SlbHLH* genes

To characterize the functions of *SlWRKY* and *SlbHLH* genes, GO enrichment analysis was conducted using the GO online tool. The results showed that the majority of the genes of both families were primarily involved in molecular functions and biological processes such as DNA binding (GO:0003677), regulation of DNA-templated transcription (GO:0006355), regulation of gene expression (GO:0010468), protein dimerization process (GO:0046983), regulation of RNA biosynthesis (GO:2001141), and nucleobase-containing compound biosynthetic process (GO:0034654) (**Figures 7C, D**).

### Expression analysis of *SlWRKY* and *SlbHLH* genes during cadmium stress

**Figure 8:**
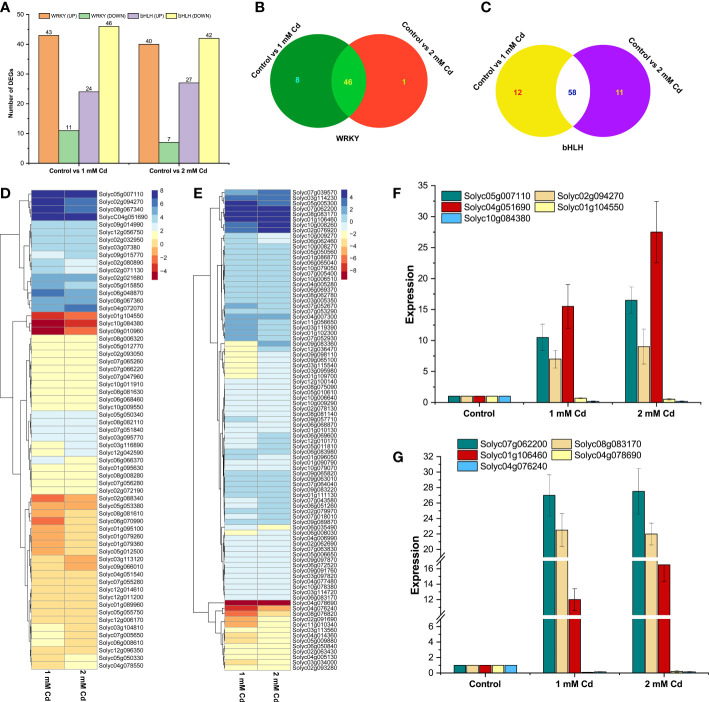
**(A)** Statistics of DEG (upregulated and downregulated) gene count comparisons of 1 and 2 mM of Cd stress with control. **(B)** Venn diagrams of the changes in differentially expressed *SlWRKY* genes in 1 and 2 mM of Cd stress vs. control. **(C)** Venn diagrams of the changes in differentially expressed *SlbHLH* genes in 1 and 2 mM of Cd stress vs. control. **(D)** Expression profiling of *SlWRKY* genes under Cd stress and **(E)** expression profiling of *SlbHLH* genes under Cd stress after DESeq2 analysis. Quantitative real-time PCR (qRT-PCR) verification of the expression level of **(F)** five *SlWRKY* genes and **(G)** five *SlbHLH* genes obtained by RNA-seq under 1 and 2 mM of Cd stress conditions. The *X*-axis represents the treatments, and the *Y*-axis shows the relative expression levels of the genes validated by qRT-PCR.

*WRKY* and *bHLH* transcription factors play an important role in plants’ heavy metal stress defense (Guo et al., 2022). However, little information is available about the Cd-responsive *WRKYs* and *bHLHs* in tomato (*S. lycopersicum*). To determine the expression levels of the target genes in tomato plants under heavy metal stress, RNA-seq analysis was taken into consideration. The overall analysis of downregulated and upregulated genes was based on the differentially expressed genes in both Cd concentrations (1 and 2 mM of Cd) (**Figures 7E, F**). Genes were downregulated and upregulated based on log_2_ fold-change >2 and log_2_ fold-change <−2, respectively, with an adjusted *p*-value <0.05. Overall, 54 and 47 *SlWRKY* genes were differentially expressed in 1 and 2 mM of Cd stress, respectively, in which 43 and 40 genes were upregulated while 11 and 7 *SlWRKY* genes were found to be downregulated (**Figure 8A**). Similarly, approximately 46 common *SlWRKYs* were differentially expressed in both 1 and 2 mM of Cd stress (**Figure 8B**). On the other hand, a total of 70 and 69 *SlbHLH* genes were differentially expressed in 1 and 2 mM of Cd stress, respectively. However, most of the *SlbHLH* genes were to be downregulated in both 1 and 2 mM of Cd stress (**Figures 8A–C**). These findings suggest significant variations in both *WRKY* and *bHLH* genes between the control and Cd stress plants (**Figures 8D, E**). As shown in **Figure 8D**, our results revealed that *SlWRKYs* have almost the same expression levels under 1 and 2 mM of cadmium stress. Cadmium stress highly enhanced the expression of the following *WRKY* genes: Solyc05g007110 (*SlWRKY76*), Solyc02g094270 (*SlWRKY38*), Solyc08g067340 (*SlWRKY46*), Solyc06g048870 (*SlWRKY19*), Solyc09g014990 (*SlWRKY33*), Solyc02g021680 (*SlWRKY35*), Solyc08g067360 (*SlWRKY45*), Solyc04g051690 (*SlWRKY51*), and Solyc04g072070 (*SlWRKY55*). Contrastingly, cadmium stress downregulated the expression of Solyc01g104550 (*SlWRKY9*), Solyc10g084380 (*SlWRKY44*), Solyc09g010960 (*SlWRKY49*), Solyc08g081610 (*SlWRKY29*), Solyc04g051540 (*SlWRKY13*), and Solyc06g070990 (*SlWRKY74*). The results demonstrated that most of the *SlbHLH* genes’ overall expression level was downregulated during 1 and 2 mM of Cd stress, respectively (**Figure 8E**). The expression pattern of a few genes, including Solyc05g005300 (*SlbHLH084*), Solyc07g062200 (*SlbHLH088*), Solyc08g083170 (*SlbHLH056*), Solyc07g052930 (*SlbHLH141*), Solyc07g039570 (*SlbHLH051*), Solyc10g008260 (*SlbHLH093*), Solyc03g114230 (*SlbHLH082*), Solyc02g076920 (*SlbHLH013*), and Solyc01g106460 (*SlbHLH007*), was found to be upregulated. On the other hand, the expression of Solyc04g078690 (*SlbHLH035*), Solyc04g076240 (*SlbHLH033*), Solyc03g118310 (*SlbHLH083*), and Solyc08g076820 (*SlbHLH146*) was found to be significantly downregulated when *S. lycopersicum* plants were treated with cadmium which shows the importance of *bHLH* transcriptional factors in Cd stress tolerance.

### Validation of *SlWRKY* and *SlbHLH* genes by RT-qPCR analyses

To check the RNA-seq data accuracy, the relative expression levels of five *SlWRKY* (Solyc05g007110, Solyc02g094270, Solyc04g051690, Solyc01g104550, and Solyc10g084380) and five *SlbHLH* (Solyc07g062200, Solyc08g083170, Solyc01g106460, Solyc04g078690, and Solyc04g076240) genes were determined using qRT-PCR with specific primers (**Table S3**). The expression patterns followed the same pattern as the RNA-seq data, and a substantial relationship was established between the RNA-seq and RT-qPCR results, demonstrating the reliability of RNA-seq data (**Figures 8F, G**).

## Discussion

The threat of increased heavy metal toxicity is expected to escalate as anthropogenic activities expand over the world (Maanan et al., 2015). Heavy metal stress can induce serious issues that affect the plant’s physiology and causes harmful effects leading to large yield loss and, through the food chain, affects consumers (Khan et al., 2015). Plants acquired different molecular and cellular adaptations to protect themselves from heavy metal toxicity (Ghori et al., 2019). However, most plants, including tomatoes, still need to be elucidated regarding their response to heavy metal stress. Various transcription factors play a vital role in response to heavy metal stress (Manara, 2012; Manzoor et al., 2022). Characterizing the core underlying regulatory network may advance our understanding of the TFs’ functions in heavy metal stress tolerance. Relatively little information is available regarding tomato *WRKY* and *bHLH* genes. The information generated from the comparative genome-wide analysis will provide insight into the identification and comprehensive functional characterization of the *WRKY* and *bHLH* gene families in *S. lycopersicum*. We provided the first genome-wide study of tomatoes’ *WRKY* and *bHLH* gene families and their possible role in heavy metal stress tolerance. In the current study, we identified 81 and 161 genes encoding *WRKY* and *bHLH* transcription factors in the *S. lycopersicum* genome, respectively. The phylogenetic relationship, subcellular locations, conserved motifs, gene structure, and exon–intron organization were executed to increase our understanding of *WRKY* and *bHLH* genes in *S. lycopersicum*, especially in heavy metal stress tolerance. The results suggested that the same phylogenetic group members were closely related during evolution. Ortholog genes are related genes with the same gene function that may have evolved through speciation processes. The occurrence of a greater number of orthologous gene pairs in the *Arabidopsis*–*S*. *lycopersicum WRKY* and *bHLH* TF families may indicate ancestral interactions between *Arabidopsis* and *S*. *lycopersicum* prior to separation during evolution. In *Arabidopsis*, AT4G01250 (*AtWRKY22*) has a close orthologous relationship with Solyc05g050340 (*SlWRKY58*) and Solyc05g050330 (*SlWRKY59*), and AT2G30250 (*AtWRKY25*) and AT4G23550 (*AtWRKY29*) have orthologous pairs with Solyc08g081630 (*SlWRKY56*) and Solyc02g080890 (*SlWRKY6*), respectively, and are expressed in the roots and leaves where they induced tolerance to Cu^2+^ stress (Li et al., 2020). The AT1G62300 (*AtWRKY6*) has an orthologous pair with Solyc06g070990 (*SlWRKY74*), which is involved to modulate heavy metal uptake (Castrillo et al., 2013). Similarly, AT2G30250 (*AtWRKY25*) and AT4G23550 (*AtWRKY29*) are orthologous partners of Solyc08g081630 (*SlWRKY56*) and Solyc02g080890 (*SlWRKY6*) and are involved in tolerance against Cu and Cd (Opdenakker et al., 2012). In the *bHLH* family, AT5G04150 (*AtbHLH101*) is an ortholog of Solyc09g097870 (*SlbHLH062*) and plays a crucial role in Fe deficiency responses (Sivitz et al., 2012). AT3G23210 (*AtbHLH34*) is an ortholog of Solyc12g088130 and positively regulates Fe homeostasis in *Arabidopsis* (Li et al., 2016). Gene structure analysis explains evolutionary processes such as duplication events (Abdullah-Zawawi et al., 2021). In the current study, two different TF orthologous gene families from *Arabidopsis* and *S. Lycopersicum* displayed various exon and intron numbers and organization of conserved motifs, implying possible roles in the diversification events of the two angiosperms. For instance, the *Arabidopsis* AT1G29280 (*AtWRKY65*) gene consists of two exons, while its counterpart ortholog, the *S. lycopersicum* Solyc10g007970 (*SlWRKY77*), contains three exons. These findings show that some TF family genes may have lost introns during evolutionary processes, resulting in functional changes in *Arabidopsis* and *S. lycopersicum*. The majority of *Arabidopsis*–*S*. *lycopersicum* orthologous gene pairs in the *WRKY* and *bHLH* TF families contain the same number of exons and conserved motifs, implying identical gene function acquisition during stable evolution (Dai et al., 2007). Functional similarities between *WRKY* and *bHLH* genes within the *S. lycopersicum* genome can be predicted by analyzing their co-expression networks. Important clues and deep insights regarding the physiological functions of the unexplored *WRKY* and *bHLH* TFs of *S. lycopersicum* were obtained from their co-expression patterns and interaction networks. This profiling can aid in predicting the functions of the uncharacterized partner. For instance, Solyc06g066370 is involved in inducing an immune response against *Botrytis cinerea* fungi in tomato plants (Liu et al., 2014) and has a strong co-expression link with uncharacterized Solyc03g095770, Solyc09g014990, Solyc03g116890, Solyc05g050300, and Solyc06g068460. The *bHLH* Solyc06g068870 gene is expressed highly in the aerial organs of tomato plants and is probably involved in stomata development (Ortega et al., 2019), while its partner Solyc08g005050 is still uncharacterized for its physiological function. Furthermore, the GO analysis revealed that the majority of these transcriptional factor genes were involved in transcriptional regulating activities such as DNA binding (GO:0003677), regulation of DNA-templated transcription (GO:0006355), and regulation of gene expression (GO:0010468). Transcriptomic studies of genome-wide RNA expression have paved the way for a comprehensive understanding of how genes are expressed in different physiological conditions. Thus, we investigated the expression profile of the *WRKY* and *bHLH* gene families in *S. lycopersicum* under 1 and 2 mM of cadmium stress conditions. Upon exposure to harmful cadmium stress, both up- and downregulation were found in the expression of most of the *WRKY* and some *bHLH* genes, suggesting that heavy metal stress can alter gene expression, which further leads to an increase in stress-protecting metabolites for the plant to cope with stress, for instance, Solyc05g007110 (*SlWRKY76*), Solyc02g094270 (*SlWRKY38*), Solyc08g067340 (*SlWRKY46*), Solyc04g051690 (*SlWRKY51*), Solyc04g072070 (*SlWRKY55*), Solyc12g056750 (*SlWRKY61*), Solyc02g071130 (*SlWRKY71*), Solyc05g015850 (*SlWRKY75*), Solyc05g005300 (*SlbHLH084*), Solyc07g062200 (*SlbHLH088*), Solyc08g083170 (*SlbHLH056*), Solyc01g106460 (*SlbHLH007*), Solyc10g008260 (*SlbHLH093*),Solyc02g076920 (*SlbHLH013*), and Solyc08g062780 (*SlbHLH089*). Our findings are consistent with previous studies that *SlWRKY76* is a putative regulator in response to various biotic and abiotic stresses (Huang et al., 2012). *SlWRKY46* suppressed the salicylic acid (SA) and jasmonic acid (JA) marker genes (Shu et al., 2021). In our investigation, we determined that the tomato *SlWRKY3* (Solyc02g088340) gene, which encodes a regulator of tolerance to osmotic stimuli (Hichri et al., 2017), was downregulated. This gene is activated by a variety of osmotic stressors, including SA. These findings are consistent with the SA analysis, which revealed a considerable reduction in Cd-stressed plants (Asaf et al., 2022). *SlWRKY51* forms a complex with the *SlJAV1* gene and suppresses JA biosynthesis (Tang et al., 2022), while the other highly expressed genes during the Cd stress have been reported for the first time in this study. In addition, previous studies have reported that the gene Solyc01g104550 (*SlWRKY9*), which is highly downregulated in Cd stress in the current study, is involved in increasing plant biomass and improved salt tolerance (Kissoudis et al., 2016) and that the Solyc09g010960 (*SlWRKY49*) responds to heat stress (Wang et al., 2020). AT1G69310 (*AtWRKY57*), the ortholog of Solyc04g072070 (*SlWRKY55*), plays a key role in the convergence of auxin signaling and jasmonic acid-mediated signaling during jasmonic acid-induced foliar senescence (Caicedo et al., 2021); AT2G46400 (*ATWRKY46*), the ortholog of Solyc12g056750 (*SlWRKY61*), mediates cold tolerance (Zhu et al., 2004); AT2G44745 (*AtWRKY13*), the ortholog of Solyc02g071130 (*SlWRKY71*), is negatively regulated during *Pectobacterium carotovorum* infiltration (Tang and Liu, 2021); and AT3G01970 (*AtWRKY45*), the ortholog of Solyc05g015850 (*SlWRKY75*), enhances tolerance to salt stress and phosphate starvation (Li et al., 2019). Similarly, in the *bHLH* family, Solyc01g086870 (*SlbHLH076*) is highly expressed during Cd stress and its ortholog in *Arabidopsis* (AT4G29100) is a defense-related gene (Vargas-Salinas et al., 2021). AT2G43140, the ortholog of Solyc08g062780 (*SlbHLH089*), is highly expressed during Cd stress, which is in agreement with previous investigations that AT2G43140 (*AtbHLH129*) is most likely to be involved in stress response (Meng et al., 2020) and that AT3G19860 (*AtbHLH121*), the ortholog of the highly expressed Solyc01g102300 (*SlbHLH006*) gene, is found to act as a key regulator of the iron-deficient signaling pathway (Ying, 2021). Based on the above analysis, it can be speculated that these upregulated *WRKY* and *bHLH* genes during Cd treatment might play a crucial role in *S. lycopersicum* in response to Cd stress. However, functional analyses still need to verify further the possible positive or negative roles of these *WKRY* and *bHLH* genes in plant responses to abiotic stresses.

## Conclusion

Several transcription factors significantly enhance resistance to heavy metal tolerance and homeostasis. However, most of the TFs are not explored for their specific physiological roles in *S. lycopersicum*. Hence, we conducted a genome-wide study of 81 *SlWRKY* and 161 *SlbHLH* genes. Phylogenetic analysis identified nine and 11 major clusters of *WRKY* and *bHLH* genes, respectively, and nine and 11 major clusters of *WRKY* and *bHLH* genes across both species (*Arabidopsis* and *S. lycopersicum*). The higher number of ortholog gene pairs in the *Arabidopsis–S. lycopersicum WRKY* and *bHLH* TFs represents their common ancestor before the taxonomic splitting of the angiosperms. Furthermore, similarity in gene structure, subcellular locations, conserved motifs, and co-expression interaction among *WRKY* and *bHLH* TFs of the *S. lycopersicum* predict their functional similarity. Overall, our findings presented a standpoint on the evolution of *WRKY* and *bHLH* TFs in *S. lycopersicum* and paved the way for additional functional characterization under heavy metal toxicity.

## Data availability statement

The data presented in the study are deposited in the National Center for Biotechnology Information (NCBI) repository, accession number PRJNA913645.

## Author contributions

IK, RJ, SA, and AL performed the analysis. SB performed the simple sequence repeats and phylogenetic analysis. K-MK and AA-H edited and drafted the manuscript. All authors contributed to the article and approved the submitted version.

## Supplementary material

Supplementary Figure 1 The most conserved common motifs of *SlWRKY* TFs family were identified by MEME database with the complete amino acids sequences. The aqua-blue colored motif signifies the *WRKY* motif.

Supplementary Figure 2 The most conserved common motifs of *SlbHLH* TFs family were identified by MEME database with the complete amino acids sequences. The aqua-blue colored motif signifies the *bHLH* motif.

Supplementary Figure 3 Phylogenetic tree of the *SlWRKY* genes with bootstrap values of the paralogous gene pairs.

Supplementary Figure 4 Phylogenetic tree of the *SlbHLH* genes with bootstrap values of the paralogous gene pairs.

Supplementary Table 1 The data of 81 *WRKY* and 161 *bHLH* genes identified in *S. lycopersicum* genome

Supplementary Table 2 The Accession IDs and STRING Identifier of the *WRKY* and *bHLH* TFs in *S. lycopersicum*

Supplementary Table 3 Genes and primers selected for RT-qPCR.
