# Supplementary material for: Genome-wide annotation and expression analysis of WRKY and bHLH transcriptional factor families reveal their involvement under cadmium stress in tomato (Solanum lycopersicum L.)
Source: Front Plant Sci. 2023 Jan 25;14:1100895. doi: 10.3389/fpls.2023.1100895 (PMC9905835; doi:10.3389/fpls.2023.1100895)
Supplement: Supplementary Figure 1 — The most conserved common motifs of SlWRKY TFs family were identified by MEME database with the complete amino acids sequences. The aqua-blue colored motif signifies the WRKY motif. [file DataSheet_1.zip › Supplementary/Table S2.docx]

| SlWRKYs | **S.No** | | **STRING Identifier** | **Accession ID** |
| --- | --- | --- | --- | --- |
|  | 1 | | SlWRKY33A | Solyc06g066370 |
|  | 2 | | WRKY70 | Solyc03g095770 |
|  | 3 | | SlWRKY40 | Solyc06g068460 |
|  | 4 | | SlWRKY30 | Solyc07g056280 |
|  | 5 | | LOC778199 | Solyc07g066220 |
|  | 6 | | WRKY75 | Solyc05g015850 |
|  | 7 | | SlWRKY30 | Solyc07g056280 |
| SlbHLHs | | 1 | ICE1a | Solyc06g068870 |
|  |  | 2 | bHLH1 | Solyc03g097820 |
|  |  | 3 | JA3 | Solyc08g076930 |
|  |  | 4 | fer | Solyc06g051550 |

**Co-expressed Proteins**
