# Supplementary material for: Genome-wide annotation and expression analysis of WRKY and bHLH transcriptional factor families reveal their involvement under cadmium stress in tomato (Solanum lycopersicum L.)
Source: Front Plant Sci. 2023 Jan 25;14:1100895. doi: 10.3389/fpls.2023.1100895 (PMC9905835; doi:10.3389/fpls.2023.1100895)
Supplement: Supplementary Figure 1 — The most conserved common motifs of SlWRKY TFs family were identified by MEME database with the complete amino acids sequences. The aqua-blue colored motif signifies the WRKY motif. [file DataSheet_1.zip › Supplementary/Table S3.docx]

**Figure S3.** Genes and primers selected for RT-qPCR.

| Name | F&R Primer | TM 0C | GC% |
| --- | --- | --- | --- |
| Solyc05g007110 | ATGGGCTTGAGCTTGGATTA  CATGTCGGTTGCAGAAAATG | 59  60 | 45  45 |
| Solyc02g094270 | ATCCGAGAGGATGCAAAATG  TTTGGAAAGGCGCTGTACTT | 60  59 | 45  45 |
| Solyc04g051690 | GGAGGTGTTGGATGATGGAT  TTGTTGGGAGGGAATTGTGT | 59  60 | 50  45 |
| Solyc01g104550 | TCCGCTACAATGAATGATGG  AATAGCAGCAGCAACAGCAA | 59  59 | 45  45 |
| Solyc10g084380 | GATCAGCGACGAGAAAAAGC  TCTTCAGAATTTCCGGCATC | 60  60 | 50  45 |
| Solyc07g062200 | CATGCAATGAAGGAGTGGAA TTTTTCCCCATCAACTTGGA | 59  60 | 45  40 |
| Solyc08g083170 | TCGGATGAGTCTTCGGCTAT  ACGTTCATCAATCTCGCACA | 59  60 | 50  45 |
| Solyc01g106460 | TTCCCGGACTCAAAAAGATG  CTGAGCGCAAATTTTTGACA | 60  59 | 45  40 |
| Solyc04g078690 | CGACTCATCCACGTTCTTCA  AAAGACCCGGGAACATATCC | 59  60 | 50  50 |
| Solyc04g076240 | ACGAACGAACCGTCAAAACT  CCAAAGCCCTATTTTCCACA | 59  59 | 45  45 |
