# Supplementary figures and images for: Genome-wide annotation and expression analysis of WRKY and bHLH transcriptional factor families reveal their involvement under cadmium stress in tomato (Solanum lycopersicum L.)
Source: Front Plant Sci. 2023 Jan 25;14:1100895. doi: 10.3389/fpls.2023.1100895 (PMC9905835; doi:10.3389/fpls.2023.1100895)

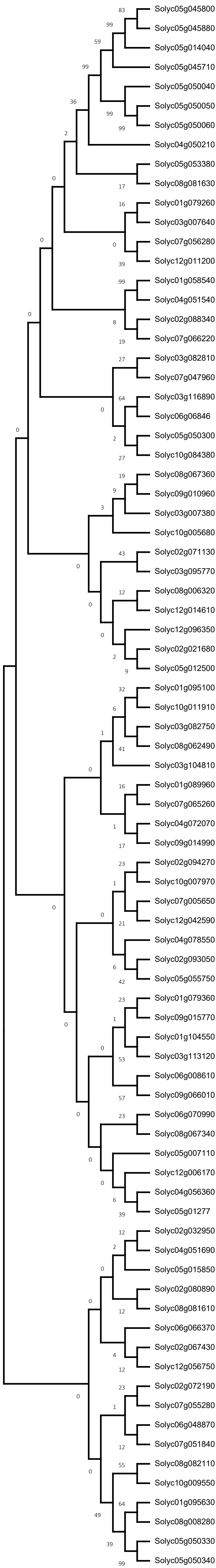

Supplement: Supplementary Figure 1 — The most conserved common motifs of SlWRKY TFs family were identified by MEME database with the complete amino acids sequences. The aqua-blue colored motif signifies the WRKY motif. [file DataSheet_1.zip › Supplementary/Figure S3.pdf]

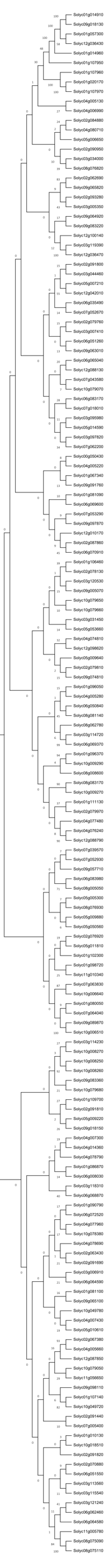

Supplement: Supplementary Figure 1 — The most conserved common motifs of SlWRKY TFs family were identified by MEME database with the complete amino acids sequences. The aqua-blue colored motif signifies the WRKY motif. [file DataSheet_1.zip › Supplementary/Figure S4.pdf]
